# An Uncommon Case of Acute Coronary Stent Thrombosis

**DOI:** 10.7759/cureus.33834

**Published:** 2023-01-16

**Authors:** Kyle Coombes, Guarina Molina, Rafael Contreras, Andrew Jacobson, Robert Jarrett

**Affiliations:** 1 School of Medicine, American University of the Caribbean, Cupecoy, SXM; 2 Internal Medicine, Danbury Hospital, Danbury, USA; 3 Cardiology, Danbury Hospital, Danbury, USA

**Keywords:** acute myocardial infarction, myocardial infarction, drug-eluting stent, drug eluting stents (des), coronary artery thrombosis, dual anti-platelet therapy, cardiovascular intervention

## Abstract

Coronary stent thrombosis is an uncommon complication of percutaneous coronary intervention, which can result in myocardial infarction and often death. We present a case of acute stent thrombosis in a patient with newly diagnosed triple vessel coronary artery disease occurring within less than an hour of stent placement along with a review of the literature.

## Introduction

Bare metal stents (BMS) were introduced in the late 1980s and have been replaced by drug-eluting stents (DES) over time due to decreased risk of myocardial infarction, cardiovascular complications, and mortality [1]. While each type of stent carries its own risks and benefits, stent thrombosis, most commonly seen in DES, remains a rare but often fatal complication. Drugs used in these stents (sirolimus, everolimus, and paclitaxel) may halt re-endothelization of smooth muscle and delay healing, inducing tissue growth expression and promoting thrombogenicity [2].

Stent thrombosis is a rare but potentially catastrophic complication of percutaneous coronary intervention (PCI), with an incidence of 0.1-1.7% and a mortality rate of over 45% [3]. While no single risk factor has been proven to predict stent thrombosis, the most consistent predictive factors include premature discontinuation of antiplatelet therapy, the extent of coronary disease, and stent number/length [4].

## Case presentation

A 36-year-old male with a past medical history of untreated hypertension and a family history of premature coronary artery disease (CAD) presented with chest pain that started early in the morning of admission. The pain was described as a “pressure-like” sensation and graded at a level 3/10 in intensity across the left upper chest and radiating to his left shoulder. He initially disregarded the symptoms and proceeded with his daily routine, which included walking his dog, when the pain suddenly intensified and was associated with nausea and dyspnea. Due to the intensity and persistence of symptoms, he took 800 mg of ibuprofen and 324 mg of aspirin before presenting to the emergency department.

**Figure 1:**
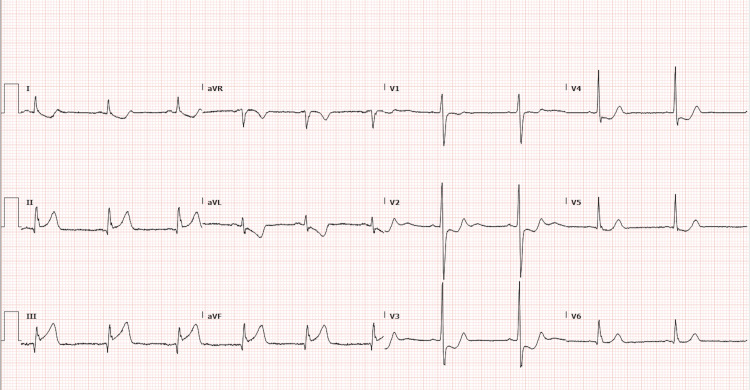
EKG from presentation ST elevations in leads II, III, and aVF (2 mm in leads II and III; 1.5 mm in aVF) with ST depressions in aVL and leads V2-V5 (avL: 1.5 mm, V2: 2 mm, V3: 2.5 mm, V4: 1.5 mm, and V5: 1 mm), consistent with acute inferoposterior ST-elevation myocardial infarction (STEMI).

On admission, vital signs showed a heart rate of 70 bpm with a respiratory rate of 20 breaths/minute, blood pressure of 121/89 mmHg, and oxygen saturation (SpO2) of 100% on room air. A thorough physical exam was unremarkable. Pertinent blood work showed a creatinine of 1.29 mg/dL (unknown baseline), fifth-generation troponin at 8,298 ng/L, total cholesterol of 262 mg/dL, low-density lipoprotein (LDL) cholesterol of 262 mg/dL, and a hemoglobin A1c of 5.3%. Initial electrocardiogram (EKG) showed ST-elevations in leads II, III, and aVF, with ST-depressions in aVL and leads V2-V5 (Figure 1), consistent with acute inferoposterior ST-elevation myocardial infarction (STEMI), requiring emergent cardiac catheterization.

**Figure 2:**
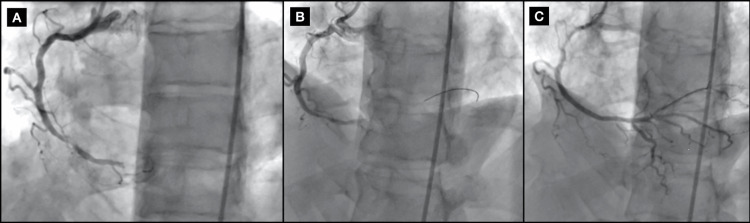
First angiogram (A) Pre-intervention, showing 100% distal occlusion of the right coronary artery (RCA) (LAO 26°/CRAN 0°). (B) Pre-intervention, showing 100% distal occlusion of the RCA with wire through lesion (LAO 26°/CRAN 18°). (C) Following drug-eluting stent (DES) placement (LAO 26°/CRAN 18°). LAO: left anterior oblique; CRAN: cranial.

**Figure 3:**
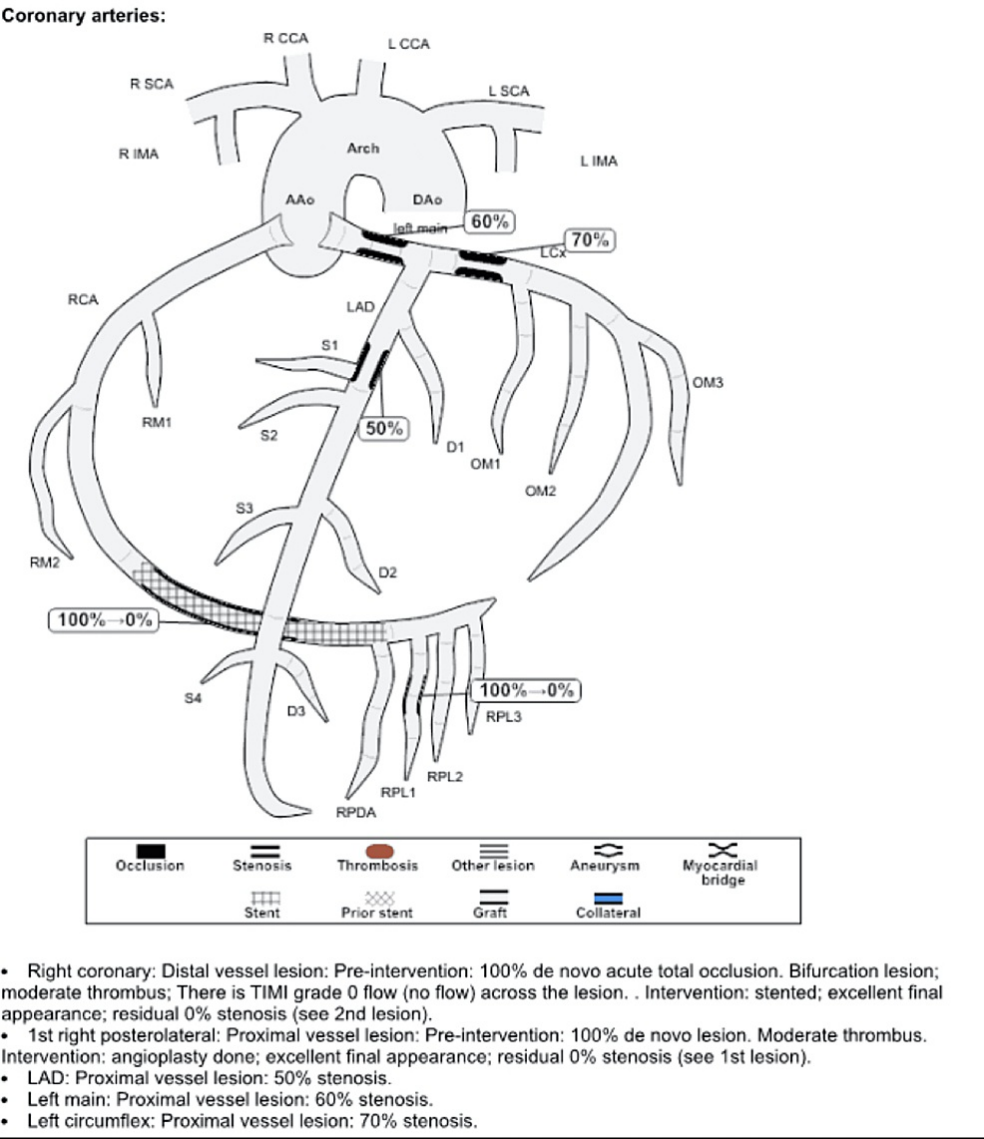
Illustration of the first angiogram RCA: right coronary artery; LAD: left anterior descending artery; LCx: left circumflex artery; TIMI: Thrombolysis in Myocardial Infarction; SCA: subclavian artery; IMA: internal mammary artery; CCA: common carotid artery; RPL: right posterolateral artery; RPDA: posterior descending coronary artery; AAo: ascending aorta; DAo: descending aorta; S (S1, S2, S3, S4): septal arteries; D (D1, D2, D3): diagonal arteries; OM (OM1, OM2, OM3): obtuse marginal artery; RM (RM1, RM2): ramus artery.

The patient underwent left heart coronary angiography and cardiac catheterization, which demonstrated triple vessel CAD with 60% proximal stenosis in the left main coronary artery (LMCA), 50% proximal stenosis in the left anterior descending artery (LAD), 70% stenosis in the left circumflex artery (LCX), and 100% bifurcate distal vessel lesion in the right coronary artery (RCA) with moderate filling defect consistent with thrombus, which was likely the culprit of his clinical presentation, as well as a 100% proximal vessel lesion in the 1st right posterolateral artery (RLP1). PCI performed in the distal RCA yielded successful placement of two everolimus-DES (EES) (2.25 mm x 38 mm and 2.75 mm x 12 mm, respectively) post-balloon angioplasty, with residual 0% stenosis with Thrombolysis in Myocardial Infarction (TIMI) grade 3 flow (Figures 2, 3). The procedure was completed without complications, with the exception of two episodes of non-sustained ventricular tachycardia, managed with an amiodarone drip.

**Figure 4:**
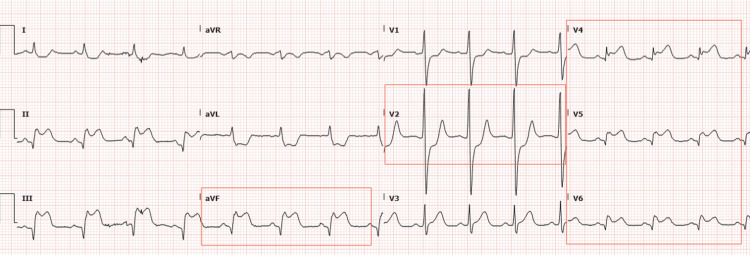
EKG post-percutaneous coronary intervention Inferolateral ST elevations and new ST elevations in anteroapical leads (3.5 mm in aVF and 1.5-2.5 mm in V4-V6).

Within 30 minutes of catheterization and stent placement, the patient presented with repeated episodes of chest pain with a new EKG showing worsening inferolateral ST elevations and new ST elevations in anteroapical leads in V4-V6 (Figure 4).

**Figure 5:**
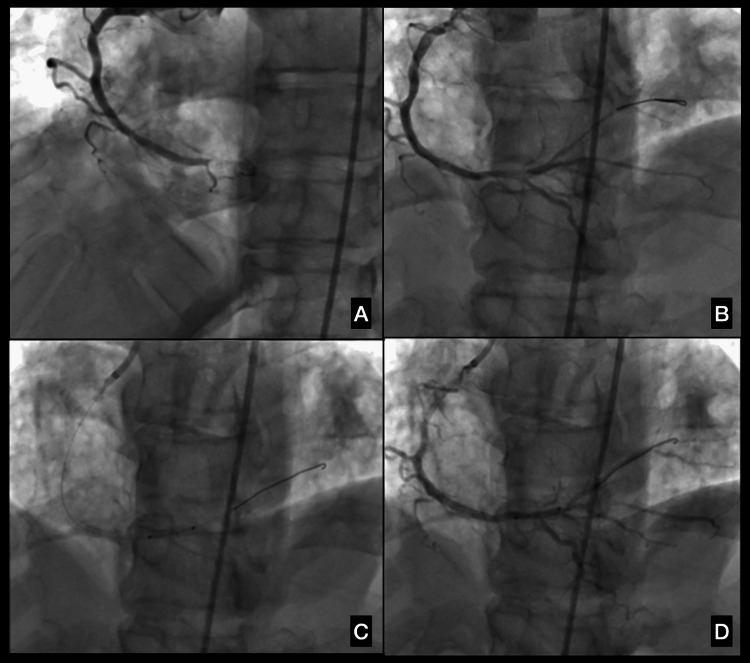
LHC after RCA stent thrombosis (A) Pre-intervention (LAO 24°/CRAN 0°). (B) Pre-intervention: wire crossing the lesion helped to break up the thrombus and restore some blood flow (LAO 24°/CRAN 17°). (C) Pre-intervention: balloon placement (LAO 24°/CRAN 17°). (D) Post-balloon inflation (LAO 24°/CRAN 17°). LHC: left heart catheterization; RCA: right coronary artery; LAO: left anterior oblique; CRAN: cranial.

**Figure 6:**
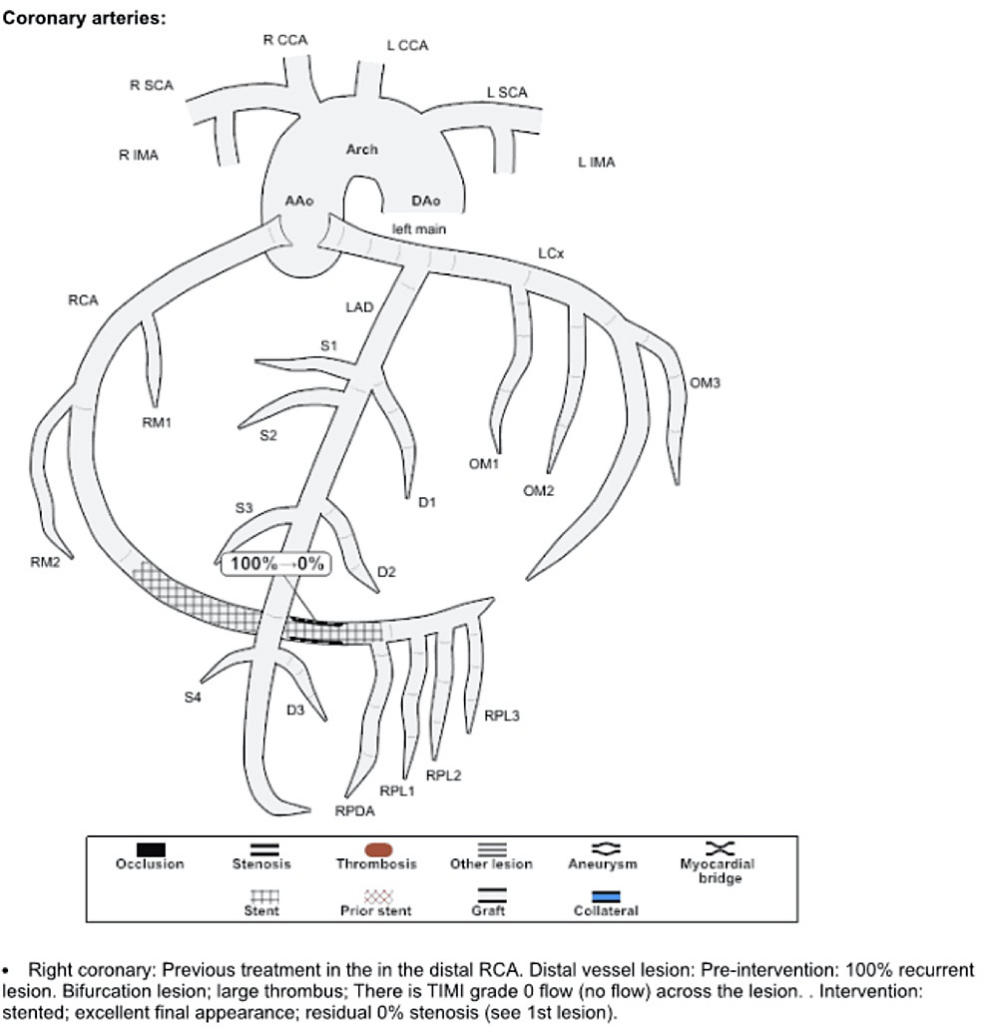
Illustration of repeat angiogram after thrombosis RCA showed distal vessel occlusion with 0% residual stenosis post re-stenting. RCA: right coronary artery; LAD: left anterior descending artery; LCx: left circumflex artery; TIMI: Thrombolysis in Myocardial Infarction; SCA: subclavian artery; IMA: internal mammary artery; CCA: common carotid artery; RPL: right posterolateral artery; RPDA: posterior descending coronary artery; AAo: ascending aorta; DAo: descending aorta; S (S1, S2, S3, S4): septal arteries; D (D1, D2, D3): diagonal arteries; OM (OM1, OM2, OM3): obtuse marginal artery; RM (RM1, RM2): ramus artery.

Given his age and acuity of symptoms in the setting of worsening EKG, a repeat angiogram (Figures 5, 6) was obtained to ensure patency of the stent, which revealed a thrombotic stent occlusion of the RCA requiring mechanical thrombectomy and re-stenting. Following the procedure, he was started on an eptifibatide drip (Integrilin) for 18 hours before being switched to dual antiplatelet therapy (DAPT) with prasugrel. Amiodarone was discontinued on hospital day two. Additional work-up with lipoprotein A and apolipoprotein B was found to be within normal limits (25 mg/dL and 116 mg/dL, respectively). Repeat echocardiogram had no significant changes in systolic function or regional wall motion abnormalities from a previous study (ejection fraction (EF) of 50-55%, akinesis of entire inferior and basal inferolateral wall, and hypokinesis of the basal-inferoseptal and mid inferolateral wall).

Upon stabilization, the patient was transferred out of the intensive care unit into the medical ward for continued care. He was discharged after a four-day stay on DAPT (aspirin and prasugrel), high-dose atorvastatin, ezetimibe, and metoprolol tartrate with outpatient follow-up with cardiology and cardiothoracic surgery. Coronary artery bypass grafting (CABG) was recommended due to the extent of vascular injury and vessel involvement. He underwent the procedure a month after discharge, requiring two grafts from the left internal mammary artery (LIMA) to LAD and left radial to ostial marginal with no recurrence of symptoms at seven months follow-up.

## Discussion

PCI is the gold standard for coronary re-vascularization through angioplasty and placement of DES or BMS. The study of DES has helped cardiologists avoid restenosis and thrombosis by releasing anti-restenotic drugs [4], with current-generation stents including EES among other agents. Despite the well-studied literature and the minimally invasive nature of the procedure, a myriad of complications of both cardiac and non-cardiac origin have to be taken into consideration.

A definite stent thrombosis is defined as an angiographic thrombus originating in the stent or within 5 mm of the stent, with or without vessel occlusion, associated with ischemic symptoms, acute changes in EKG, or changes in cardiac biomarkers. The timeframe begins once the patient has been undraped and removed from the catheterization laboratory. According to the Academic Research Consortium (ARC) revision, an acute stent thrombosis must occur within the first 24 hours after stent implantation (0.4% of cases) [3]. Other time frames include subacute (24 hours to 30 days), late (31 days to one year), and very late (>one year) (incidence of 1%, 0.4%, and 0.5%, respectively) [3,5]. In the case of our patient, occlusion and manifestation of symptoms occurred within 30 minutes of placement.

Overall, stent thrombosis is characterized by platelet activation and aggregation, but causes are multifactorial and may include reduced left ventricular ejection fraction, malignancy, smoking, or factors concerning the procedure itself, such as stent malapposition or length, and dissections [6]. Other causative factors for thrombosis include small vessel lesions, chronic occlusive lesions, intramural hematomas, or, in the case of our patient, bifurcation of lesions. Regardless, premature discontinuation of DAPT remains the front-runner for stent thrombosis [6].

DAPT relies on the synergy of cyclooxygenase-1 inhibitors (aspirin) and P2Y12 receptor inhibitors (clopidogrel, prasugrel, or ticagrelor) to prevent clot formation [7]. The length of DAPT depends on the stability of ischemic heart disease, the type of stent used, and the bleeding risk. Patients with acute coronary syndrome (ACS) have a recommended period of at least 12 months with aspirin and P2Y12-inhibitor therapy; whereas a shorter duration is feasible in patients with a history of bleeding [8,9]. Premature discontinuation of DAPT, particularly at six months in those with DES, with ACS and no overt signs of bleeding, represents an increased risk for myocardial infarction [10,11].

If necessary, therapy prolongation can be assessed with risk-assessment scales such as the PRECISE-DAPT score [12]. In 2000, the Clopidogrel Aspirin Stent International Cooperative Study (CLASSICS) showed the new drug clopidogrel to be superior to ticlopidine when combined with aspirin to prevent cardiac events post-stenting [13]. However, more recent studies have shown limitations such as irreversibility and hepatic conversion from pro-drug to active metabolite [8]. The approval of more novel P2Y2 inhibitors, such as prasugrel and ticagrelor, has increased the challenges and risk assessment when selecting an adequate agent to protect from ischemic events while balancing an acceptable bleeding risk. In the case of our patient, prasugrel was chosen given the patient’s age and superiority over other agents at reducing the one-year incidence of ischemic events without significant bleeding risks [13].

## Conclusions

Restenosis and occlusion of the coronary stent remain a complication of PCI despite years of investigation and, in some cases, no evident risk factors for occlusion. While the mechanisms of injury may include patient characteristics or direct complications of the procedure, these may develop at any stage after the procedure and at this time there is no fault-proof method to prevent it. In the meantime, DAPT remains the mainstay of therapy for preventing stent thrombosis, but its efficacy is also based on patient characteristics as well as compliance. While studies may show minor advantages for particular P2Y12 agents, analysis on efficacy and safety among the three agents show equal mortality rates at one year.
